# Variant detection sensitivity and biases in whole genome and exome sequencing

**DOI:** 10.1186/1471-2105-15-247

**Published:** 2014-07-19

**Authors:** Alison M Meynert, Morad Ansari, David R FitzPatrick, Martin S Taylor

**Affiliations:** MRC Human Genetics Unit, MRC Institute of Genetics and Molecular Medicine, University of Edinburgh, Western General Hospital, Crewe Road, EH4 2XU Edinburgh, UK

**Keywords:** SNP, Sensitivity, Protein-coding genes, Next-generation sequencing, Whole genome sequencing, Exome sequencing

## Abstract

**Background:**

Less than two percent of the human genome is protein coding, yet that small fraction harbours the majority of known disease causing mutations. Despite rapidly falling whole genome sequencing (WGS) costs, much research and increasingly the clinical use of sequence data is likely to remain focused on the protein coding exome. We set out to quantify and understand how WGS compares with the targeted capture and sequencing of the exome (exome-seq), for the specific purpose of identifying single nucleotide polymorphisms (SNPs) in exome targeted regions.

**Results:**

We have compared polymorphism detection sensitivity and systematic biases using a set of tissue samples that have been subject to both deep exome and whole genome sequencing. The scoring of detection sensitivity was based on sequence down sampling and reference to a set of gold-standard SNP calls for each sample. Despite evidence of incremental improvements in exome capture technology over time, whole genome sequencing has greater uniformity of sequence read coverage and reduced biases in the detection of non-reference alleles than exome-seq. Exome-seq achieves 95% SNP detection sensitivity at a mean on-target depth of 40 reads, whereas WGS only requires a mean of 14 reads. Known disease causing mutations are not biased towards easy or hard to sequence areas of the genome for either exome-seq or WGS.

**Conclusions:**

From an economic perspective, WGS is at parity with exome-seq for variant detection in the targeted coding regions. WGS offers benefits in uniformity of read coverage and more balanced allele ratio calls, both of which can in most cases be offset by deeper exome-seq, with the caveat that some exome-seq targets will never achieve sufficient mapped read depth for variant detection due to technical difficulties or probe failures. As WGS is intrinsically richer data that can provide insight into polymorphisms outside coding regions and reveal genomic rearrangements, it is likely to progressively replace exome-seq for many applications.

**Electronic supplementary material:**

The online version of this article (doi:10.1186/1471-2105-15-247) contains supplementary material, which is available to authorized users.

## Background

The cost of sequencing DNA has decreased steeply since the introduction of next-generation short read technologies [1]. It is now at the point where cohorts of whole human genomes are sequenced for study. However, investigations of disease-causing variation continue to focus on the protein-coding exome, which is a small fraction of the whole genome. It contains fewer repetitive elements than non-coding regions and contains most of the causal disease variants identified to date [2]. Additionally, experimental approaches to determine the function of candidate disease variants at protein coding or transcript splice sites are well developed and accepted by the research community.

For these reasons, exome centric analysis will remain common in research and is increasingly used in clinical genetic settings [3]. The targeted capture followed by sequencing of specific regions, such as the 30 Mb human exome (exome-seq), has proven to be a cost-effective and productive strategy for the identification of single nucleotide polymorphisms (SNPs) and small insertions and deletions in this rich vein of the genome. However, as sequencing technology rapidly improves and cost per sequenced nucleotide falls, there is likely to come a point where it is more economic to sequence a whole genome rather than target-capture and sequence, even if analysis is confined to just the exome. Where that point lies depends both on the costs of the technologies but also on the uniformity of coverage and biases inherent in the data. In this work we set out to compare exome-seq with whole genome sequencing (WGS) in terms of their sensitivity to correctly detect known variants over the whole exome.

The process of exome-seq has known issues that impact negatively on SNP detection sensitivity. These include PCR amplification, which tends towards lower coverage in GC-rich regions due to annealing during amplification [4–6], and the preferential capture of reference sequence alleles, which biases the allele distribution away from alternate alleles at heterozygous SNP sites [7–9]. Exome-seq produces a relatively heterogeneous profile of read coverage over target regions when compared to the more homogeneous WGS [10]. Better uniformity of coverage yields improved SNP detection sensitivity across the regions of interest [9–11].

Previous estimates of the amount of sequencing required to accurately identify SNPs in WGS and exome-seq are variable. Bentley *et al.* estimated that 15X mapped read depth of WGS samples would be sufficient to detect almost all homozygous SNPs and 33X for almost all heterozygous SNPs [12]. 50X was estimated by Ajay *et al.* for all SNPs and small indels [13]. Depending on the capture kit, Clark *et al.* calculated that exome-seq required 80X mean on-target depth to reach the common threshold of 10X per-site depth in 90% or more of all targeted regions [10]. Our previous work on some of the original exome-seq target capture kits estimated between 20X and 46X mean on-target depth was required to successfully genotype 95% of heterozygous SNPs, with the commercially available kits at the higher end of that range.

**Figure 1:**
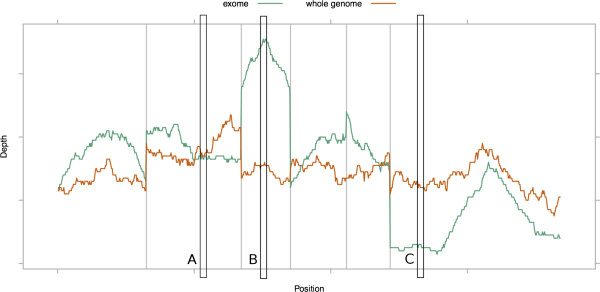
**SNP detection sensitivity in exome and whole genome sequencing.** Exome and whole genome sequencing mapped read depth across the exons of an example gene. The grey vertical lines denote exon boundaries. At point A, the depth is equal and we can compare the per-site SNP detection sensitivity. Points B and C are examples of unequal depth, where per-site sensitivity cannot be directly compared, but the overall estimated sensitivity of the region can be calculated to account for the variability in coverage.

We examine previously established measures of SNP detection sensitivity [9] in coding regions from exome-seq and WGS samples. SNP detection sensitivity can be measured both at a site level, considering the number of reads mapped over a given position in the reference genome, or as an overall estimate based on the mapped read depth across a region or regions (Figure 1). We computed the per-site measure for different sequencing technologies and compared them directly when the per-site mapped depth is identical (point A, Figure 1). Because of the allele distribution bias in exome-seq, we expected that WGS would require fewer reads to successfully genotype heterozygous SNPs. The greater variability in coverage from exome-seq means that greater mean on-target depth should be required to identify the same proportion of SNPs in exome-seq as compared to WGS [10] (points B and C, Figure 1). We measured the estimated overall SNP detection sensitivity across a given set of target regions by using the per-site SNP detection sensitivity for the sequencing method combined with the coverage distribution for samples sequenced by the same method. This relates the overall sensitivity of a method to the mean on-target depth in the sample, which can be used to calculate the cost of sequencing to a given sensitivity.

## Results and discussion

### Site level SNP detection sensitivity

Site level SNP detection sensitivity is the mapped read depth directly over a polymorphic site that is required to reliably call that polymorphism [9]. Ten human whole genome sequences (TCGA-WGS) and matched whole exome sequences from the same patients, plus ten additional exome samples (all TCGA-WXS), were obtained from The Cancer Genome Atlas (TCGA; only non-tumour samples were used). A further six human whole genome samples (1KG-WGS) were obtained from the 1000 Genomes Project [14], all aligned to the reference genome. An additional 13 exome samples were captured, sequenced, and aligned in house as part of two ongoing disease studies (HGU-WXS) (Methods, Additional file 1: Table S1, Additional file 2: Tables S2–S4, Additional file 1: Figures S1 and S2). We randomly downsampled all 49 alignments to simulate shallower sequencing and called SNPs in the coding regions of the alignments as in our previous work [9].

**Figure 2:**
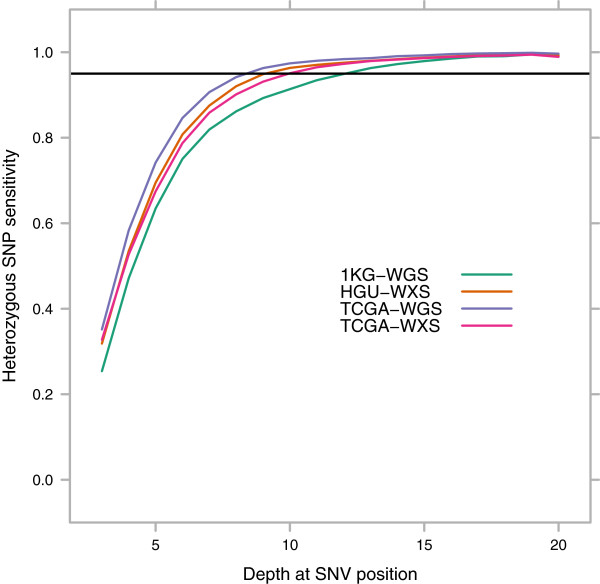
**Site level heterozygous SNP detection sensitivity for exome and whole genome sequencing samples.** Sensitivity is calculated from heterozygous HapMap 3.3 positions [16] located within coding sequence as determined by Ensembl 72 [17]. 95% sensitivity is reached at per-site mapped depths of 9X for the TCGA-WGS samples, 10X for the TCGA-WXS and HGU-WXS samples, and 13X for the 1KG-WGS samples.

We defined a gold-standard set of SNP calls for each sample, based on the full alignments (using all available reads for the sample, i.e. not down sampled) and confined to known HapMap 3.3 variants (Additional file 2: Table S5, Additional file 1: Figure S3, Additional file 3). We validated the use of HapMap 3.3 variants as the gold standard in sample NA12878 by comparing results to those obtained using the Genome in a Bottle 2.18 highly confident variant call set [15] as the gold standard (Additional file 1: Figure S4). We measured sensitivity as a function of the per-site depth for heterozygous (Figure 2) and homozygous (not shown) SNPs. We focused on heterozygous SNPs as the more challenging problem: only 2-3X per-site depth was required to accurately detect at least 95% of homozygous SNPs in all four data sets.

The oldest data set (1KG-WGS) had the worst performance for heterozygous SNPs, requiring at least 13X to reach 95% sensitivity. This could be due to shorter read lengths or higher sequencing error rates on older technologies. The difference in per-site SNP detection sensitivity between this data set and the newer three data sets indicates that analysis of older data sets requires more stringent thresholds.

All of the newer three data sets, which are contemporary with each other, performed similarly. The TCGA-WXS samples reached 95% sensitivity at 10X, while the HGU-WXS and TCGA-WGS samples had a slight edge at 9X. There was a slight advantage in sensitivity for the newer TCGA-WGS data set as opposed to the two exome-seq data sets, though this equalized at 12-13X per-site depth. Given that read lengths are the same between these three data sets and most samples were sequenced on the same generation of machine, this difference was likely caused by reference bias from the capture step. Low-depth exome sequencing projects may need to take this into consideration.

All the data sets converged at 99% sensitivity for sites with between 15 and 20X mapped depth. The recall curves were virtually identical for variants drawn from the Ensembl 72 coding regions and each of the sets of targeted regions from the two exome sequencing data sets (Additional file 1: Figure S5, Additional file 1: Table S6). Specificity (1 - false positive rate) reached 99% at 9X for the 1KG-WGS data set and 8X for the other three (Additional file 1: Figure S6).

#### Direct comparison of matched samples

The matched WGS and exome-seq samples from TCGA were compared directly. Considering only SNPs in the regions targeted by the exome capture kit, 98.3 ± 0.007% of variant sites were called as polymorphic by both methods with matched genotypes, and 0.3 ± 0.3% with mismatched genotypes. A further 1.3 ± 0.4% were called as polymorphic by whole genome sequencing only, and 0.1 ± 0.1% by exome sequencing only. 93% of the sites called as polymorphic only by whole genome sequencing had greater mapped per-site depth in the WGS sample than in the exome-seq sample (Additional file 1: Figure S7). Of these, 34% are at sites with no reads in the exome-seq sample, which could be due to probe failure or other technical problems.

**Figure 3:**
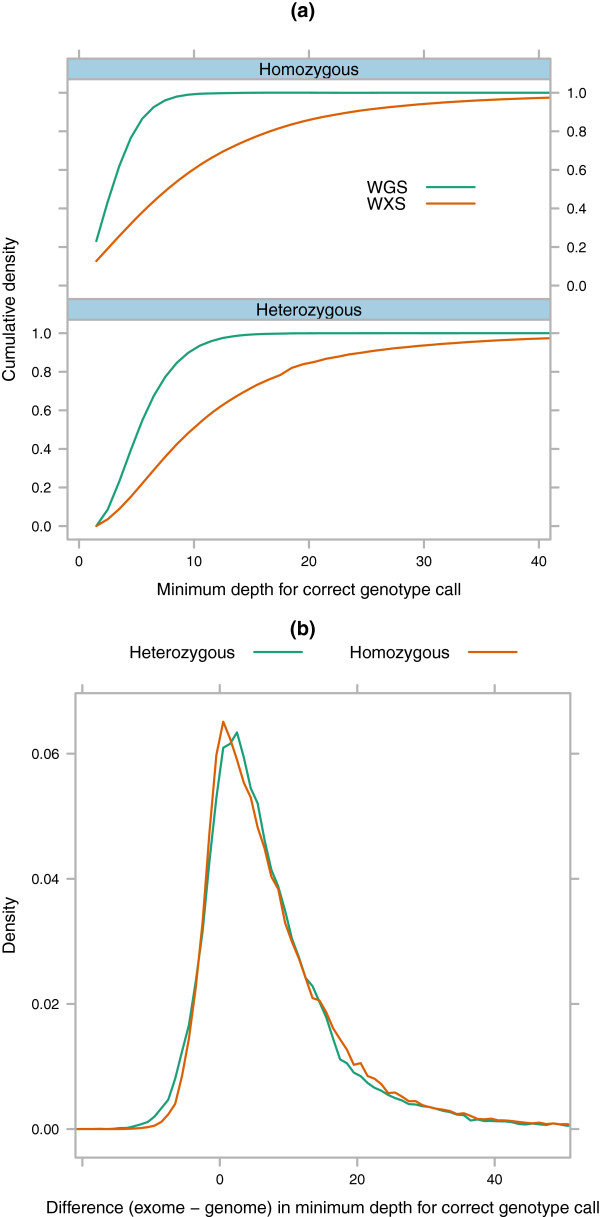
**Minimum per-site mapped depth required to correctly genotype a site in matched TCGA exome and genome samples.** Coding SNPs at HapMap 3.3 positions [16] with identical genotypes and alleles between matched TCGA exome and genome samples in the full alignments. **a)** The minimum per-site mapped depth required for a correct genotype call in TCGA-WXS and TCGA-WGS matchd samples. **b)** The number of extra reads required to correctly identify a SNP in the TCGA-WXS sample.

The majority of mismatched genotypes were cases where the whole genome sample was genotyped as heterozygous and the exome sample was genotyped as homozygous (Additional file 1: Figure 8a and Table S7). Mismatches generally occurred at sites where the whole genome sample had higher per-site mapped depth than the exome sample (Additional file 1: Figure S8b). Some sites with very high mapped depth in the exome sample also had mismatched genotypes with the whole genome sample. This could be caused by random accumulation of the same sequencing error at a given position if that position is sequenced to very high mapped depth. Both of these results imply that improving uniformity of coverage will improve SNP detection sensitivity.We took the subset of coding SNPs where the alleles and genotypes were identical in the full alignments between the TCGA-WGS and TCGA-WXS samples for the same individual, and compared the mapped depth of sequencing required to correctly identify the genotypes of both heterozygous and homozygous SNPs (Figure 3). To accurately genotype 95% of heterozygous SNPs, the TCGA-WGS data set required a minimum per-site depth of 12X and the TCGA-WXS data set required 34X. For homozygous sites, the minimum per-site depths were 8X and 33X respectively.

#### Effect of grouped and single sample variant calling

The results in this paper are derived from calling variants for one sample at a time; however, it is standard practice to call variants in groups of samples (pooled calling) as this improves accuracy by allowing the use of reads across all samples at a position to determine the presence of a polymorphism. To investigate the relative benefits of pooled calling, we grouped our samples by data source, called variants on the full alignments for each group, and compared the results to the variants called on the full alignments by single sample calling (Additional file 2: Table S8a).

For sites in HapMap 3.3, there were very few cases of mismatched genotypes between the two calling methods; the main difference was in additional sites called as polymorphic when the samples were grouped. For all data sets, of the sites with mismatched genotypes or where only one method called the site as polymorphic, the mapped read depth was on average lower than for sites where genotypes were matched (Additional file 1: Figure S9). The two exome capture data sets benefited significantly from grouped sample calling, with a mean of 186 (594) heterozygous and 100 (326) homozygous additional sites genotyped for the HGU-WXS (TCGA-WXS) data set. These data sets had the most samples, which may have been the major cause of the improvement, or possibly the uneven coverage of the exomes was smoothed by the inclusion of multiple samples. The 1KG-WGS data set also benefited to the same degree as the exome capture data sets for heterozygous sites (mean 317 additional), but not for homozygous sites (mean 52 additional), perhaps because the 1KG-WGS data set comprises two family trios, which would help to resolve heterozygous positions.

The number of variants called from the TCGA-WGS data set did not improve greatly with grouped sample calling (mean of 37 heterozygous and 11 homozygous additional sites), though there were a large number of mismatched genotypes between the group calling and the single-sample calling. This was observed in only 6 of the samples; the other 4 all had ≤ 2 mismatched genotypes. The TCGA-WGS samples had both excellent mean on-target depth and uniformity of coverage, which made them easy to accurately genotype using single-sample variant calling. Grouped variant calling would therefore not provide the same boost as with the other data sets.

We also examined rare variants, as defined by absence from HapMap 3.3, presence in the Exome Variant Server ESP6500 (http://evs.gs.washington.edu/EVS) set at less than 0.01 minor allele frequency, and minimum genotype quality at least 60 in each of the grouped and single sample call sets (Additional file 2: Table S8b). The 1KG-WGS, HGU-WXS, TCGA-WGS, and TCGA-WXS data sets gained a mean of an additional 0.28%, 0.25%, 1.23%, and 4.30% respectively of these rare variants by grouped calling, while losing only 0.31%, 0.04%, 0.19%, and 0.35% that were only called in the single sample method. The TCGA-WXS data set gained by far the most rare variants by use of the grouped calling, similar to the results for known common HapMap 3.3 sites.

### Overall estimated sensitivity

**Figure 4:**
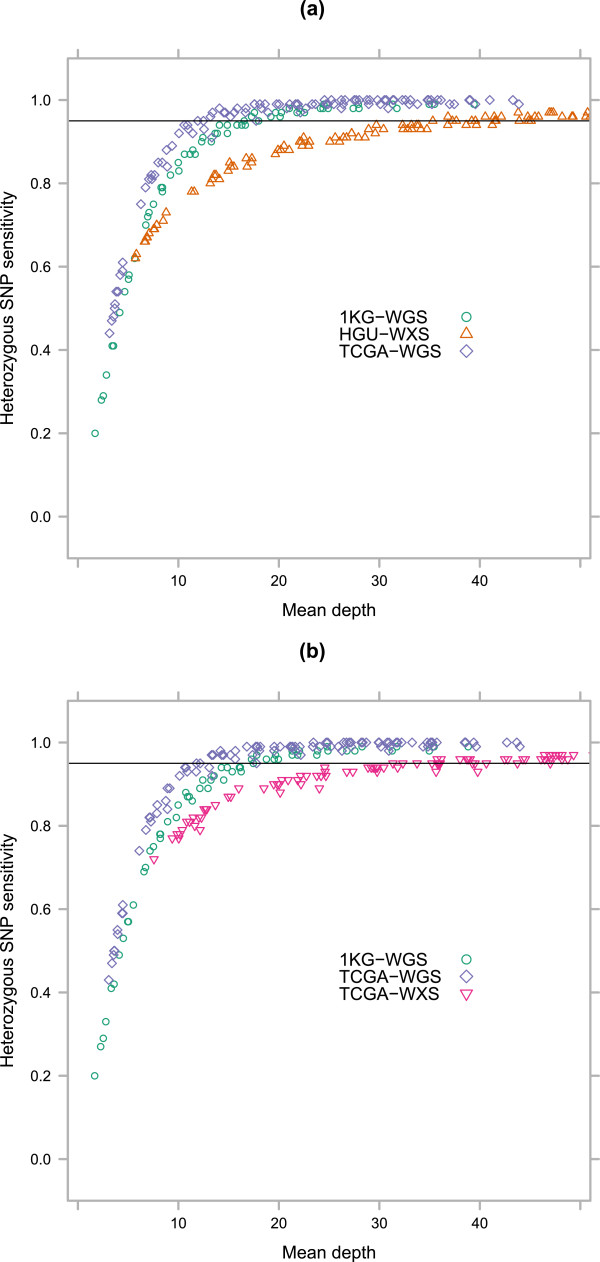
**Overall estimated sensitivity for targeted regions.** Calculated from the per-site sensitivity for each data set combined with the depth of coverage distributions for samples across the regions targeted by each of the two exome capture kits. **a)** HGU-WXS (Nimblegen SeqCap EZ Exome v3). **b)** TCGA-WXS (Whole exome Agilent 1.1 plus boosters).

Using the depth of coverage distributions for every downsampled and full alignment on the regions targeted by each of the two exome capture kits, and the per-site SNP detection sensitivity for each data set, we calculated the overall estimated sensitivity for each of the four data sets. We compared this measure to the mean on-target read depth across the targeted regions and found that the two whole genome data sets performed considerably better than each of the exome data sets (Figure 4). In order to reach an overall estimated 95% sensitivity for heterozygous SNPs in the targeted regions, the 1KG-WGS samples required at least 18X and the TCGA-WGS 14X mean on-target depth. The HGU-WXS samples required 41X mean on-target depth, and the TCGA-WXS samples 39X. This effect is almost entirely due to the lack of uniformity in coverage for the exome samples: The difference in per-site sensitivity is relatively slight between the two exome data sets and the TCGA-WGS data set (Figure 2), and both of them perform better than the 1KG-WGS data set on that measure.

Our estimates for WGS required mapped depth are lower than those from Bentley *et al.* (33X) [12] and Ajay *et al.* (50X) [13], though both were attempting to quantify detection of all or almost all SNPs rather than to a given percentage as here. It is unsurprising that the harder to sequence variants will require proportionally greater additional numbers of reads to accurately genotype. Additionally, we are analysing only coding sequence variants, which are in the least repetitive portion of the human genome. The higher figures reported by the other WGS analyses will be influenced by the different qualities of non-coding sequence, especially repetitive regions. The TCGA-WXS and HGU-WXS exome-seq data sets used in this analysis can update the figures provided by Clark *et al.* of 80X mean on-target depth required for 10X mapped read depth in 90% of targeted regions [10]: a median of 59X mean on-target depth is needed for the same coverage in both of our more recent exome-seq data sets. The equivalent figure was 18X for the TCGA-WGS data set and 20X for the 1KG-WGS data set.

#### Sensitivity at sites in Human Gene Mutation Database (HGMD)

Overall estimated sensitivity is a useful measure that can be applied to more specific subsets of target regions. For instance, estimating how many known disease causing or disease associated SNV sites can be recovered given a particular sequencing strategy. To demonstrate and at the same time discover if known disease causing mutations are preferentially located in easy or difficult to sequence regions of the genome: we obtained the locations of such coding and splice variants from HGMD [18]. From these we generated the coverage distributions for disease-causing and disease-associated SNVs separately to compare their overall estimated sensitivity with coding regions in general for both whole genome and exome sequencing. For 87,663 disease-causing and 2,241 disease-associated sites, we found no difference in the measure across all four of our sample sets (Additional file 1: Figure S10).

#### Characteristics of difficult target regions

As has previously been noted for both whole genome and exome sequencing, regions of high G+C content and regions containing repetitive elements are generally harder to sequence to high depth [19]. We define difficult regions based on poor coverage (see Methods) in at least half the samples from a given data set, and easy regions based on excellent coverage (see Methods) in all the samples from that set. Our samples show the expected characteristics, with the bulk of difficult regions occurring at G+C content above 60% (Additional file 1: Figure S11), and with a significantly higher proportion of difficult regions overlapping repetitive elements compared to relatively easy target regions (Additional file 1: Figure S12). The HGU-WXS data set also had a large number of difficult target regions that were of low G+C content. Because the classification of a region as difficult is based on at least half the samples in a data set, this was not caused by capture failure of one or a few samples; however, a larger scale failure could be implicated. Very few target regions were classed as difficult for the TCGA-WGS data set for either of the two exome capture target region sets. However, approximately one third of all regions identified as difficult in any of the four data sets were classed that way for both the TCGA-WXS and 1KG-WGS data set, and 15% for both the HGU-WXS and the 1KG-WGS data set (Additional file 1: Figure S13).

To quantify the contributions of repetitive sequence and nucleotide composition to target difficulty, we identified targets meeting our criteria for difficult (see Methods) in any of the samples for a data set. The number of samples in which that target was defined as difficult was multiply regressed against target G+C content, presence of annotated repeats and alignability [20]. All factors were significant to *p*<0.001; however, their predictive power was slight (Additional file 1: Table S8). The adjusted R-squareds were 0.265, 0.086, 0.150, and 0.171 for 1KG-WGS, HGU-WXS, TCGA-WGS, and TCGA-WXS respectively. As the analysis was run on the intersection of the target capture regions for the two exome-seq data sets; the particularly low R-squared for the HGU-WXS data set may be due to differences in probe design but is not due to a different set of targets.

### Cost of sequencing to a given level of sensitivity

We compared exome and whole genome sequencing costs on current standard technology (Illumina HiSeq) with an exome capture kit of the same size as the Nimblegen SeqCap EZ Exome v3 (65Mbp) used for the HGU-WXS samples, assuming 60% of exome reads on target (Table 1) and holding the per sample cost of the exome capture kit constant. To achieve 93–94% overall estimated heterozygous sensitivity in the coding regions of the genome, exome sequencing is 4.2X cheaper than whole genome sequencing (12 exome samples/lane vs. 1 lane/sample whole genome). Likewise, for 98–99% sensitivity, exome sequencing is 5.4X cheaper (4 exome samples/lane vs. 2 lanes/sample whole genome).

**Table 1:** **Cost of sequencing to achieve a given level of heterozygous SNV detection sensitivity**

Method	Lane usage	Mean on-target depth	Sensitivity	Cost
Whole genome	1 lane/sample	11X	94.0%	4.60
	2 lanes/sample	22X	98.5%	8.79
Exome	16 samples/lane	22X	91.1%	1.00
	12 samples/lane	29X	93.4%	1.09
	8 samples/lane	44X	95.9%	1.28
	6 samples/lane	58X	96.9%	1.46
	4 samples/lane	88X	98.1%	1.63

All costs have been normalised against the cheapest exome sequencing (16 samples per lane). Estimated costs include library preparation, exome capture and multiplexing where applicable, and paired-end sequencing on Illumina HiSeq.

We estimate that the cost per lane of sequence would have to be 15–20% of the current cost for the two methods to reach cost parity, holding the cost of exome capture constant (Additional file 1: Figure S14). The projected $1000 genome at 30X depth enabled by the Illumina HiSeq X Ten (X10) system reaches this cost point for 93–94% overall estimated heterozygous sensitivity in the coding regions of the genome. Holding the per sample exome kit cost constant, the X10 system claims to sequence genomes to 12X depth at 77% the cost of sequencing exomes to 29X depth, with roughly equivalent sensitivity. However, for higher sensitivity of 98–99%, we estimate that WGS on the X10 system will still be 31% more expensive than exome-seq, and decreases in exome capture kit costs will likely keep the two methods at close to cost parity.

## Conclusions

Exome-seq target capture technology is clearly improving. Our previous results from a solution-based target capture kit suggested a mean on-target depth of 46X was needed to obtain 95% overall estimated sensitivity for heterozygous SNPs [9]. The two data sets in this analysis from more recent capture kits (HGU-WXS and TCGA-WXS) show 40X is required for the same level of sensitivity. This progressive improvement in technology could partially explain the difference between our results and the higher mean on-target depth of 80X suggested by other previous analyses such as Clark *et al.* [10].

The mean on-target depth needed for 95% SNP detection sensitivity shown by our analysis of WGS data from multiple sources is also lower than previous estimates [12, 13]. The earlier of these two estimates describes reads from the first next-generation sequencing experiments, which were shorter than the reads used for our WGS samples, and additionally contained no paired-end reads. The second estimate is more comparable in terms of data, and we conclude that improvement in variant calling algorithms is likely to be a factor in the difference here.

Uniformity of coverage is clearly still a major issue for exome sequencing in terms of capturing a reasonable number of reads across all of the targeted regions. PCR amplification-free library preparation can mitigate the issue somewhat for WGS samples [4, 5] but it is still required to provide a sufficiently large library for exome-seq samples. Allele distribution biases introduced by the reference bias of exome-seq target probes could be minimised by the use of alternate probes containing common haplotypes, but the problem will remain for rare variants. The additional allele distribution bias introduced by treating the reference genome as truth during computational analysis affects both WGS and again exome-seq and is not easily fixed for rare variants.

The amount of raw sequencing is the main cost driver for both WGS and exome-seq, and the drop in cost to the $1000 human genome at 30X depth has brought the two methods roughly into parity. However, smaller sequencing centres relying on the previous generation of machines will continue to charge three to four times exome-seq costs for the same level of SNP detection sensitivity across coding regions using WGS. When taking into account the considerably higher data storage requirements of WGS and the extra compute time required to perform alignment and subsequent bioinformatic analyses on WGS samples, the cost difference will be further amplified.

WGS provides a much richer data set, capturing information on polymorphisms over whole genome and potentially capturing genomic rearrangements. The dramatically improved uniformity of read coverage and reduced bias of allele ratios in WGS, both lend themselves to improved detection of copy number changes and measurement of sample heterogeneity. These are likely to be extremely useful measures in some settings, such as for the sequencing of primary tumours whose analysis, even when focused on the exome, is confounded by copy number change, sample heterogeneity and a desire to detect *de novo* mutations.

## Methods

### Exome capture and sequencing

The 13 HGU-WXS exomes were captured using a Nimblegen SeqCap EZ Exome v3 kit. Paired-end reads of 98 bp were generated on the Illumina HiSeq platform. Six whole genome samples were downloaded from the 1000 Genomes Project [14] Pilot 2 (high coverage family trios).

Individuals with both whole genome and exome samples from TCGA were filtered for cases with similar numbers of reads to the 1KG-WGS and HGU-WXS exome samples. From these, 20 individuals with exome samples labelled with the same set of target capture regions were randomly selected. Exome alignments for all 20 individuals and whole genome alignments for a random subset of 10 individuals were downloaded from TCGA Data Portal. Additional file 1: Table S1 summarises the technology used to generate each of the four data sets.

### Ethical approval and consent

The samples used for in-house exome sequencing were collected under approval by the UK Multiregional Ethics Committee (References: 06/MRE00/76 and 04/MRE00/19).

### Alignments

Reads for the 13 HGU-WXS exomes were aligned to the hg19/GRCh37 assembly of the human genome reference sequence with BWA 0.5.9 [21]. Duplicate reads were removed using the MarkDuplicates function of Picard 1.79 (http://picard.sourceforge.net). Reads were re-aligned around indels and quality scores re-calibrated using the Genome Analysis Toolkit (GATK) 2.2-8-gec077cd [22]. Full parameters are given in the Additional file 1: Supplementary Information and Additional file 4. We randomly down-sampled reads from exome alignments using Picard DownsampleSam, which maintains read pair information. The probability of sampling each read varied from 0.1 to 0.9 at intervals of 0.1.

### Target regions

We defined a common set of target regions using the coding regions of exons from Ensembl 72 [17]. The coding regions were merged so that every position was represented only once, 50bp of flanking sequence added to each resulting region, and the regions merged again. We also used the provided targets for the Nimblegen SeqCap EZ Exome v3 kit, a set of targets labelled ‘Whole exome Agilent 1.1 RefSeq plus 3 boosters’ obtained directly from TCGA, and a merged set of these two targets.

Mapped read depth across all the target regions was calculated using the DepthOfCoverage tool from GATK 2.6-5-gba531bd [22]. The target regions were split into maximally 100bp non-overlapping tiles for further analysis, with small tiles at target region edges. We defined difficult target region tiles as those with fewer than 50% of their bases covered at least 15X in the full alignments for at least half of the samples in a given data set, and easy target region tiles as those with all their bases covered at least 15X in the full alignments for all the samples in a given data set. G+C content for target regions was obtained using the GCContentByInterval tool from GATK GenomeAnalysisTK-2.5-2-gf57256b, and repeat element occurrences were mapped from Ensembl 73. HGMD disease causing mutations were obtained from the HGMD Professional database (March 2013 release).

Multiple linear regression of number of samples where a target region had less than 50% of bases covered at least 15X in the full alignment was performed using R *lm* on factors G+C content, presence of repeats, and alignability [20] with no interactions, where at least one sample met the criteria for a given data set. Alignability tracks were downloaded from the UCSC Genome Browser [23]. 36mer alignability was used for 1KG-WGS samples, 75mer for TCGA-WXS, and 100mer for HGU-WXS and TCGA-WGS, to best match the read lengths for each data set (Additional file 1: Table S1).

### Variants

Variants were called on the full and down-sampled alignments using the GATK 2.6-5-gba531bd UnifiedGenotyper tool [24], one sample at a time (full parameters in Additional file 1: Supplementary Information). We obtained HapMap Phase III sites and genotypes from the project FTP site [16]. Variants from this set were mapped by position and alleles to called variants in the full and down-sampled alignments. Additional variant calls were made by grouping samples by data source and running the UnifiedGenotyper tool on the full alignments for samples within each group.

### Sensitivity

Sensitivity for per-site mapped read depth and estimated overall sensitivity were calculated as in [9], using sites in HapMap 3.3 and the Ensembl 72 coding regions with minimum genotype quality of 60 in the full alignments.

## Availability of supporting data

The TCGA-WXS and TCGA-WGS data are available through dbGaP and the Cancer Genome Hub (http://cghub.ucsc.edu/) and 1KG-WGS data from the 1000 Genomes Project (http://www.1000genomes.org/data), see Additional file 1: Table S2 for accession identifiers. Reads for the HGU-WXS exome sequence data are available upon request. VCF files containing the HapMap 3.3 SNP sites and associated genotype calls and read depths in the downsampled and full alignments used to generate the main results in this article are included as additional files. The command lines for producing the downsampled alignments and calling variants are included as a plain text additional file.

## Electronic supplementary material

Additional file 1: **Supplementary Information.** Supplementary methods, figures, and small tables. (PDF 495 KB)

Additional file 2: **Supplementary Tables.** Large supplementary tables. (XLS 50 KB)

Additional file 3: **VCF files of HapMap 3.3 coding sites and genotypes.** (ZIP 53 MB)

Additional file 4: **Command lines.** (TXT 4 KB)

## Abbreviations

1KG: 1000 Genomes Project
HGU: Human Genetics Unit
GATK: Genome Analysis ToolKit
TCGA: The Cancer Genome Atlas
TCGA-WXS: TCGA Whole eXome Sequence
TCGA-WGS: TCGA Whole genome sequence.
