# Supplementary material for: Variant detection sensitivity and biases in whole genome and exome sequencing
Source: BMC Bioinformatics. 2014 Jul 19;15(1):247. doi: 10.1186/1471-2105-15-247 (PMC4122774; doi:10.1186/1471-2105-15-247)
Supplement: Supplementary file 1 — Additional file 1: Supplementary Information. Supplementary methods, figures, and small tables. (PDF 495 KB) [file 12859_2014_6520_MOESM1_ESM.pdf]

Software commands and parameters

Alignment of HGU-WXS paired-end runs with BWA 0.5.9

```
bwa aln -q 30 hg19.fasta sample_run_1.fastq > sample_run_1.sai
bwa aln -q 30 hg19.fasta sample_run_2.fastq > sample_run_2.sai
bwa sampe hg19.fasta sample_run_1.sai sample_run_2.sai
    sample_run_1.fastq sample_run_2.fastq | gzip > sample_run.bwa.sam.gz
```

Refine HGU-WXS alignments with Picard 1.79 and GATK 2.2-8-gec077cd

Remove duplicates, re-align around indels, re-calibrate quality scores

```
java -Xmx4g -jar MarkDuplicates.jar
    INPUT=sample.pre.rmdup.bam
    OUTPUT=sample.bam
    METRICS_FILE=sample.rmdup.metrics.txt
    ASSUME_SORTED=TRUE
    VALIDATION_STRINGENCY=SILENT

java -Xmx16g -jar GenomeAnalysisTK.jar
    -T RealignerTargetCreator
    -R hg19.fasta
    -known 1000G_phase1.indels.hg19.vcf
    -known Mills_and_1000G_gold_standard.indels.hg19.vcf
    -o sample.intervals
    -I sample.bam

java -Xmx16g -jar GenomeAnalysisTK.jar
    -T IndelRealigner
    -R hg19.fasta
    -known 1000G_phase1.indels.hg19.vcf
    -known Mills_and_1000G_gold_standard.indels.hg19.vcf
    -targetIntervals sample.intervals
    -I sample.bam
    -o sample.realigned.bam

java -Xmx16g -jar GenomeAnalysisTK.jar
    -T BaseRecalibrator
    -R hg19.fasta
    -knownSites dbsnp_137.hg19.vcf
    -I sample.realigned.bam
    -o sample.recal.grp

java -Xmx16g -jar GenomeAnalysisTK.jar
    -T PrintReads
    -R hg19.fasta
    -BQSR sample.recal.grp
    -I sample.realigned.bam
    -o sample.recal.bam
```

Down-sampling BAM files with Picard 1.79

Duplicates are re-marked to handle cases where reads are no longer duplicates after down-sampling.

```
for p from 0.1 to 0.9:
    java -Xmx4g -jar DownsampleSam.jar
        INPUT=sample.recal.bam
        OUTPUT=sample.$p.pre.rmdup.bam
        PROBABILITY=$p
        R=null

    java -Xmx4g -jar MarkDuplicates.jar
        INPUT=sample.$p.pre.rmdup.bam
        OUTPUT=sample.$p.bam
        METRICS_FILE=sample.$p.rmdup.metrics.txt
        ASSUME_SORTED=TRUE
        VALIDATION_STRINGENCY=SILENT
```

Depth of coverage with GATK 2.6-5-gba531bd

Target region BED files were obtained directly from The Cancer Genome Atlas (TCGA) for the TCGA-WXS samples and from the Nimblegen SeqCap EZ Exome v3 kit CD for the HGU-WXS samples.

```
java -Xmx4g -jar GenomeAnalysisTK.jar
    -T DepthOfCoverage
    -R hg19.fasta # or grch37.fasta as appropriate
    -I sample.bam
    -L targets.bed
    -omitDepthOutputAtEachBase
    -o sample.depths.tsv
```

Variant calling in targeted regions with GATK 2.6-5-gba531bd

```
java -Xmx4g -jar GenomeAnalysisTK.jar
    -T UnifiedGenotyper
    -R hg19.fasta
    -L targets.bed
    --dbsnp dbsnp_137.hg19.vcf # or b37.vcf as appropriate
    -I sample.bam
    -o sample.vcf
    -stand_call_conf 30
    -stand_emit_conf 10
    -rf BadCigar
    -glm BOTH
```

Additional File 1: Figure S1: Averaged mapped on-target depth (X) across the longest transcripts of Ensembl 72 protein-coding genes with at least three exons.

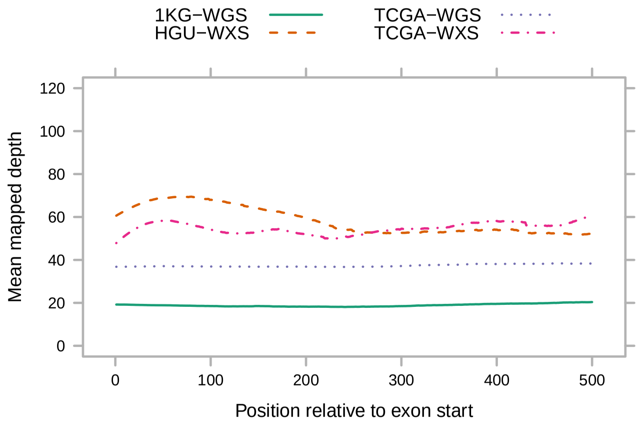

(a) First 500bp of first exons.

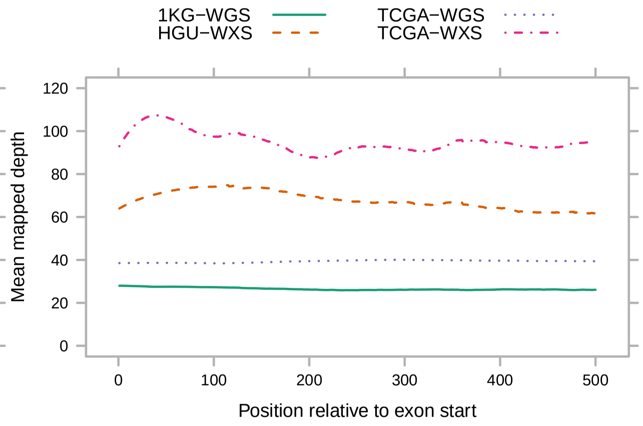

(b) First 500bp of second exons.

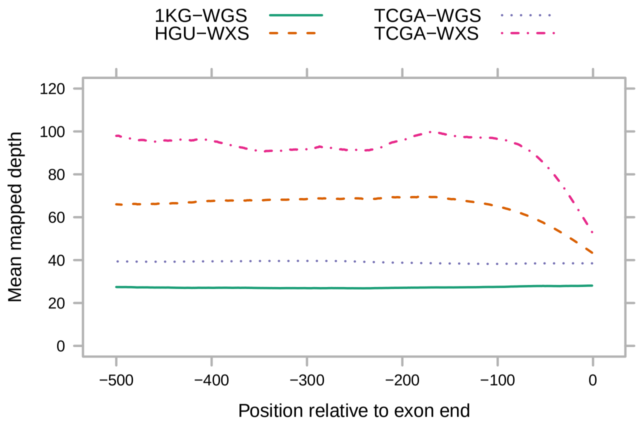

(c) Last 500bp of last exons.

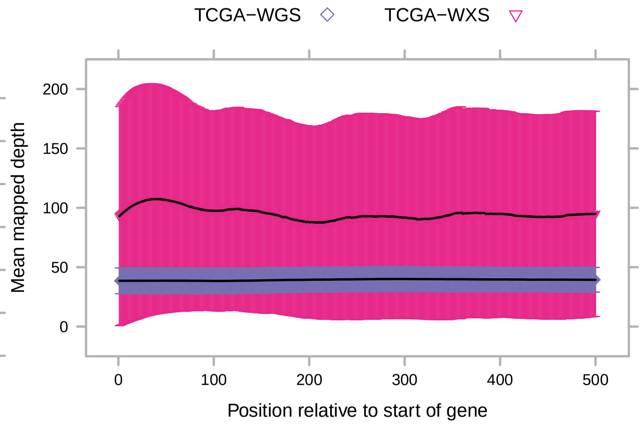

(d) First 500bp of second exons showing one standard deviation for TCGA-WXS and TCGA-WGS.

Additional File 1: Figure S2: Mean on-target depth (X) vs. raw depth (bp) for all samples in the four data sets. The target for the two whole genome data sets is the set of Ensembl 72 coding regions, and the appropriate set of target capture regions is used for each of the two exome-seq data sets.

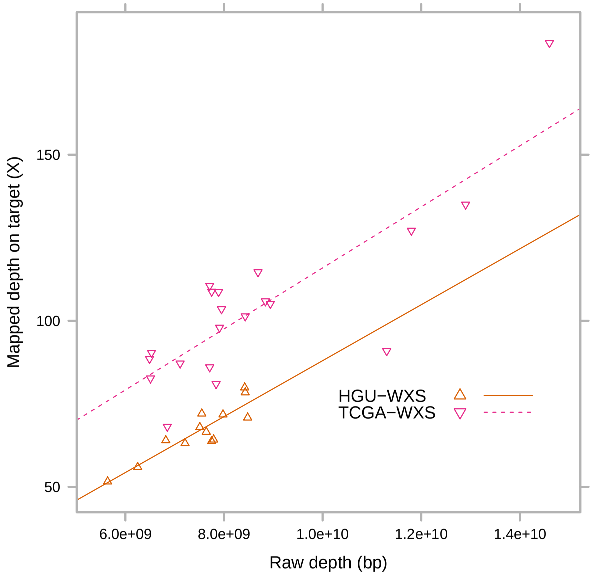

(a) Exome-seq data sets

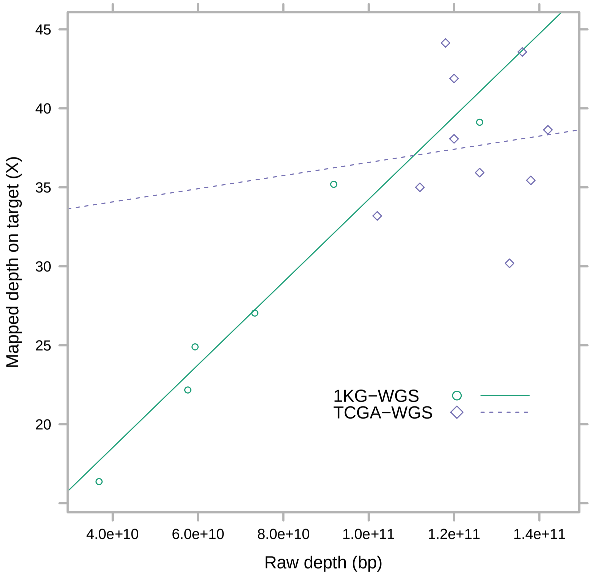

(b) Whole genome data sets

Additional File 1: Figure S3: Heterozygous and homozygous HapMap 3.3 coding SNPs in Ensembl 72 coding regions.

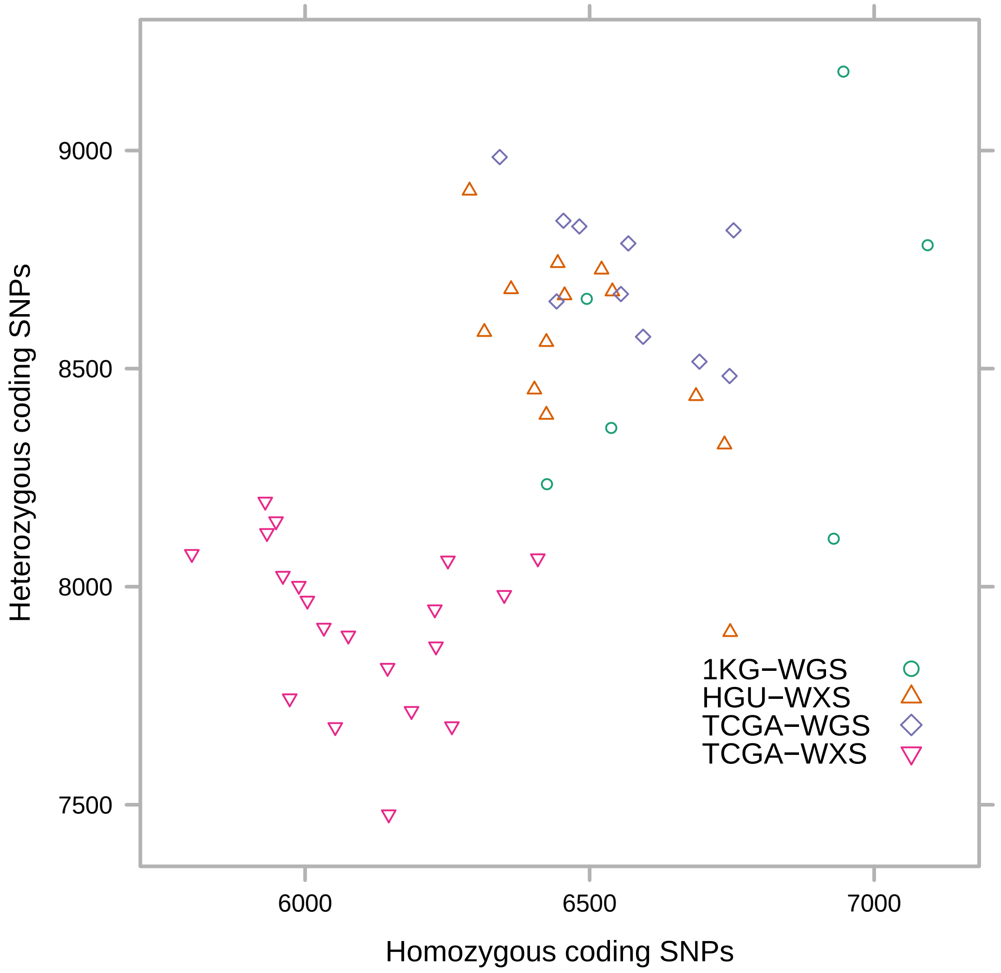

Additional File 1: Figure S4: Site level SNP detection sensitivity for sites in NA12878 in either HapMap 3.3 or Genome in a Bottle 2.18 National Institute of Standards and Technology (NIST) highly confident variant calls overlapping the Ensembl 72 coding regions. The black horizontal line indicates 95% sensitivity. 11,702 of the 15,197 HapMap 3.3 site and 16,141 Genome in a Bottle 2.18 sites were the same.

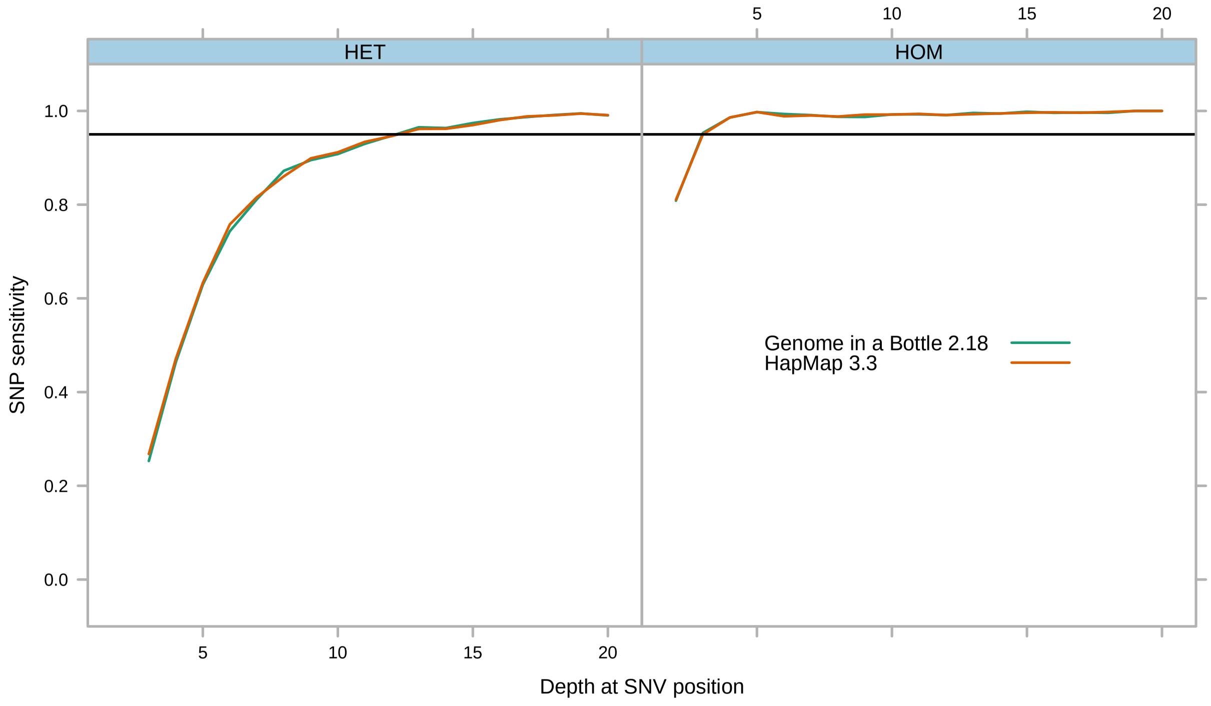

Additional File 1: Figure S5: Site level heterozygous SNP detection sensitivity for exome and whole genome sequencing samples. Sensitivity is calculated from heterozygous HapMap 3.3 positions located within coding sequence as determined by Ensembl 72, and for the subsets of these positions that are also located within the targeted regions of each of the two exome sequencing data sets. The black horizontal line indicates 95% sensitivity. The mean square difference between the curves (up to 50X) is given in Supplementary Table 5.

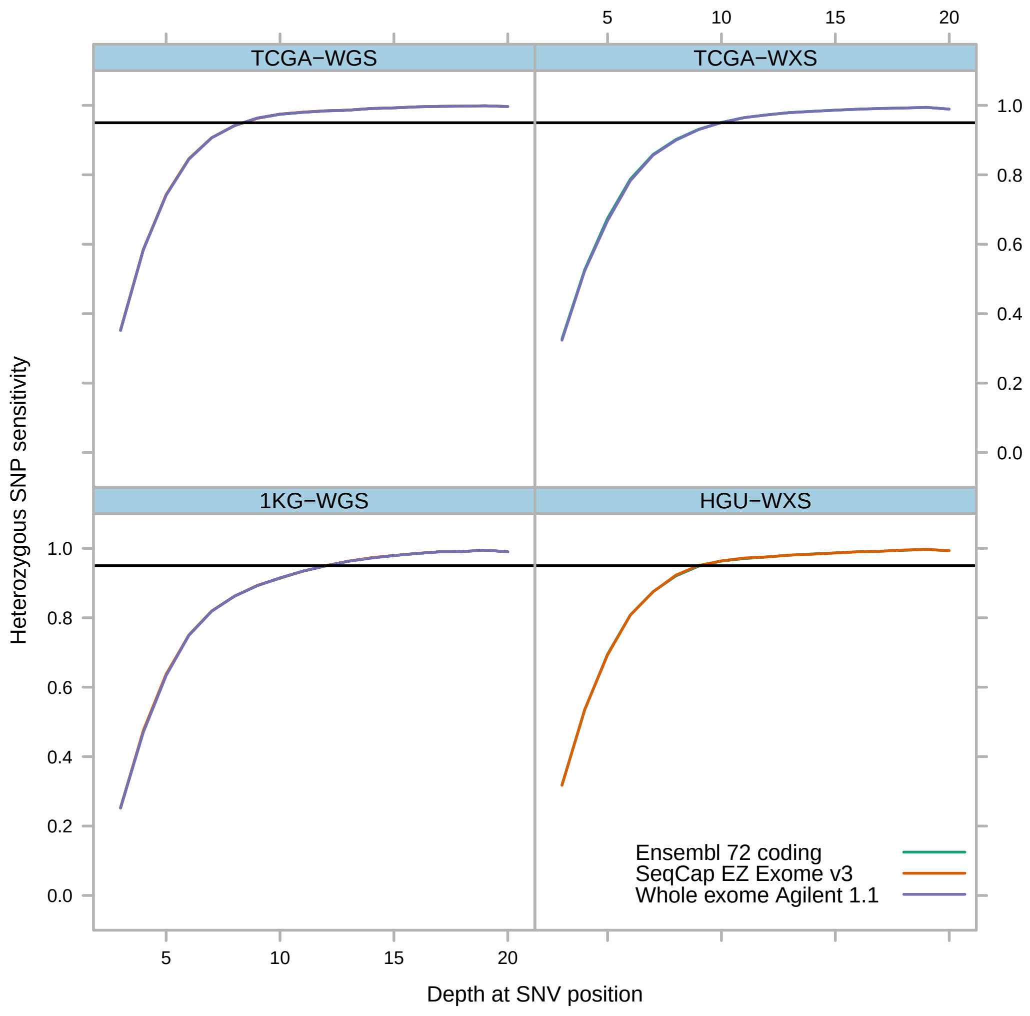

Additional File 1: Figure S6: Site level heterozygous SNP detection specificity for exome and whole genome sequencing samples. Specificity is calculated as the number of sites with concordant genotypes divided by the total of the number of sites with concordant genotypes, discordant genotypes, and false positives, using the same data sets as for calculating sensitivity. The black line horizontal line indicates 99% specificity.

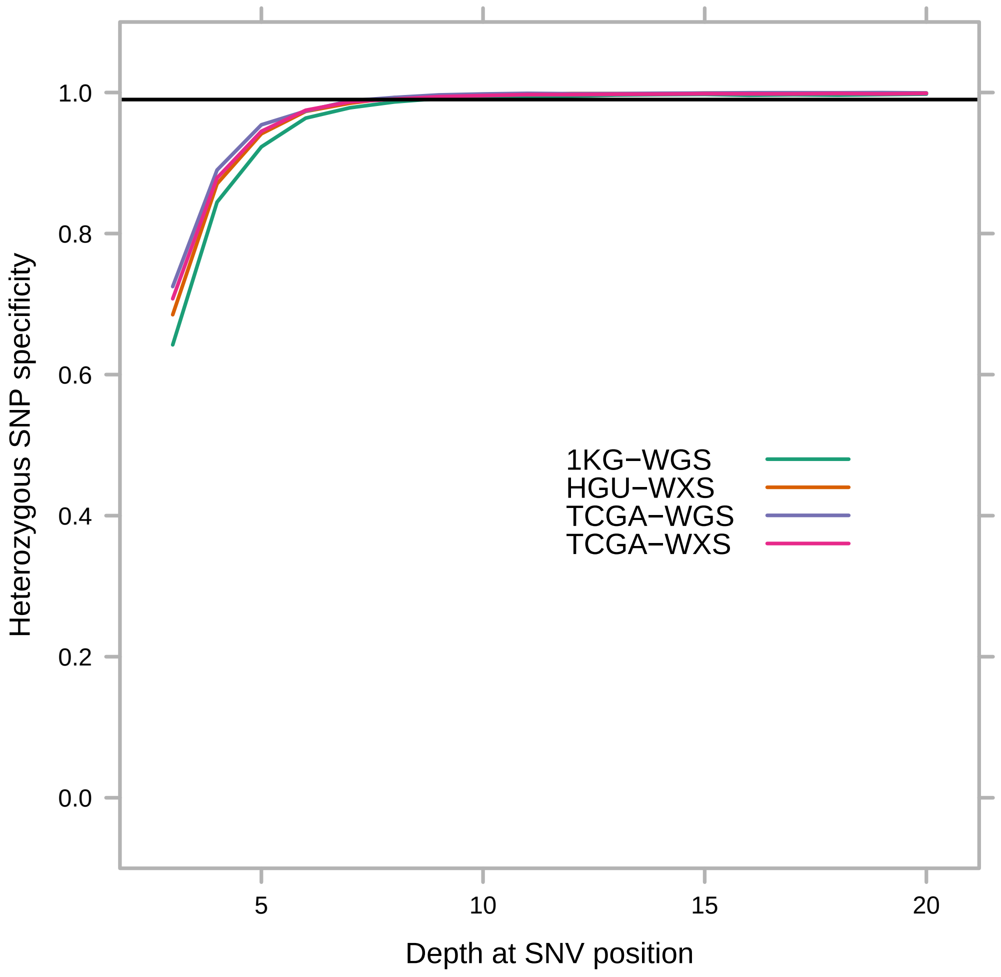

Additional File 1: Figure S7: Per-site mapped depth difference for sites found in only one of the WGS or exome-seq samples for a TCGA individual. The difference is between the sample where the site was found and the one where it was not found.

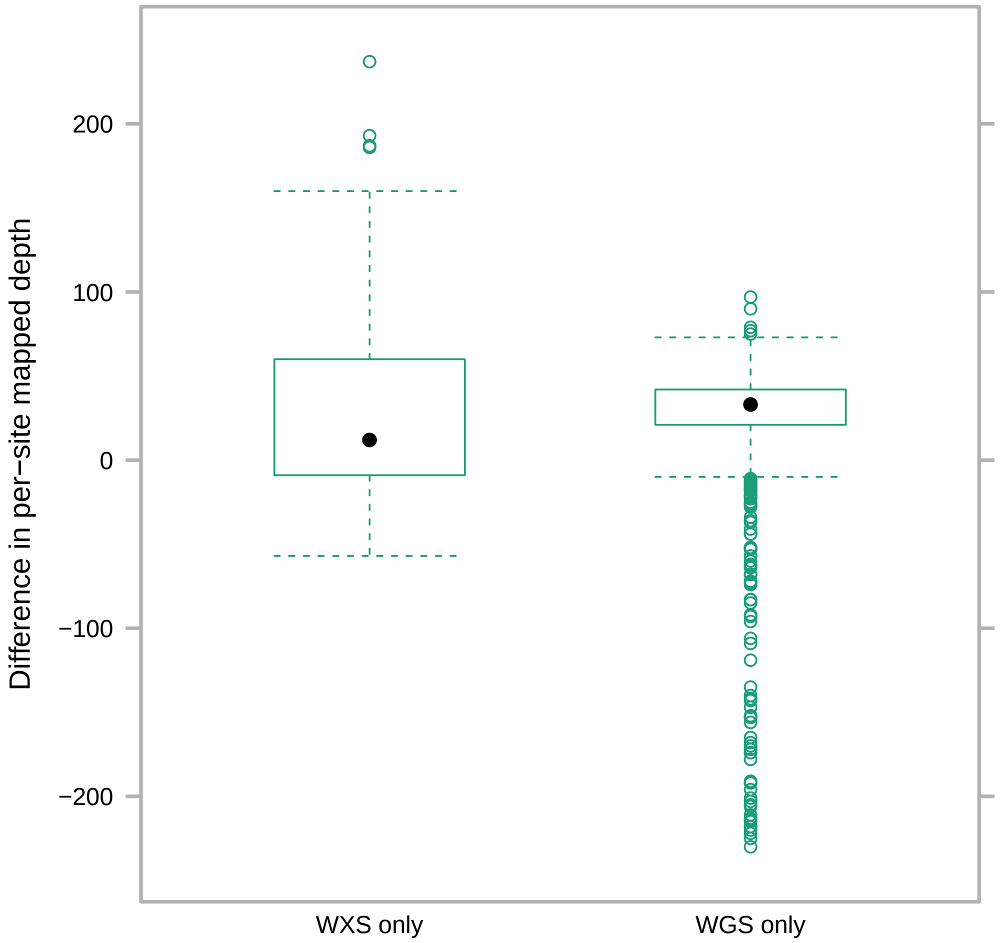

Additional File 1: Figure S8: Mismatched genotypes between matched TCGA exome and genome samples for Ensembl 72 coding SNPs at HapMap 3.3 sites.

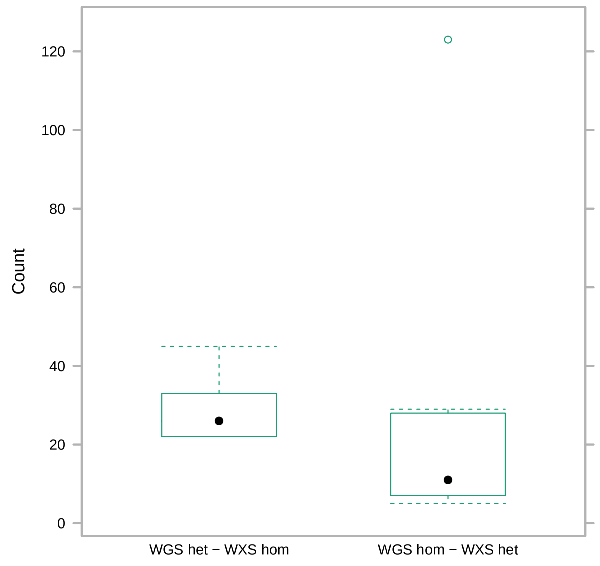

(a) Count distributions.

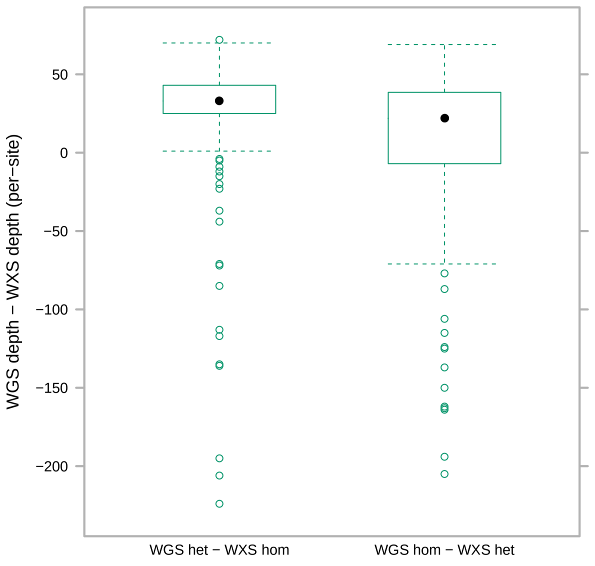

(b) Per-site mapped depth difference distributions.

Additional File 1: Figure S9: Per-site mapped depth for HapMap 3.3 sites in Ensembl 72 coding regions for grouped and single sample calling, as a proportion of median per-site mapped depth for matched sites in each data set.

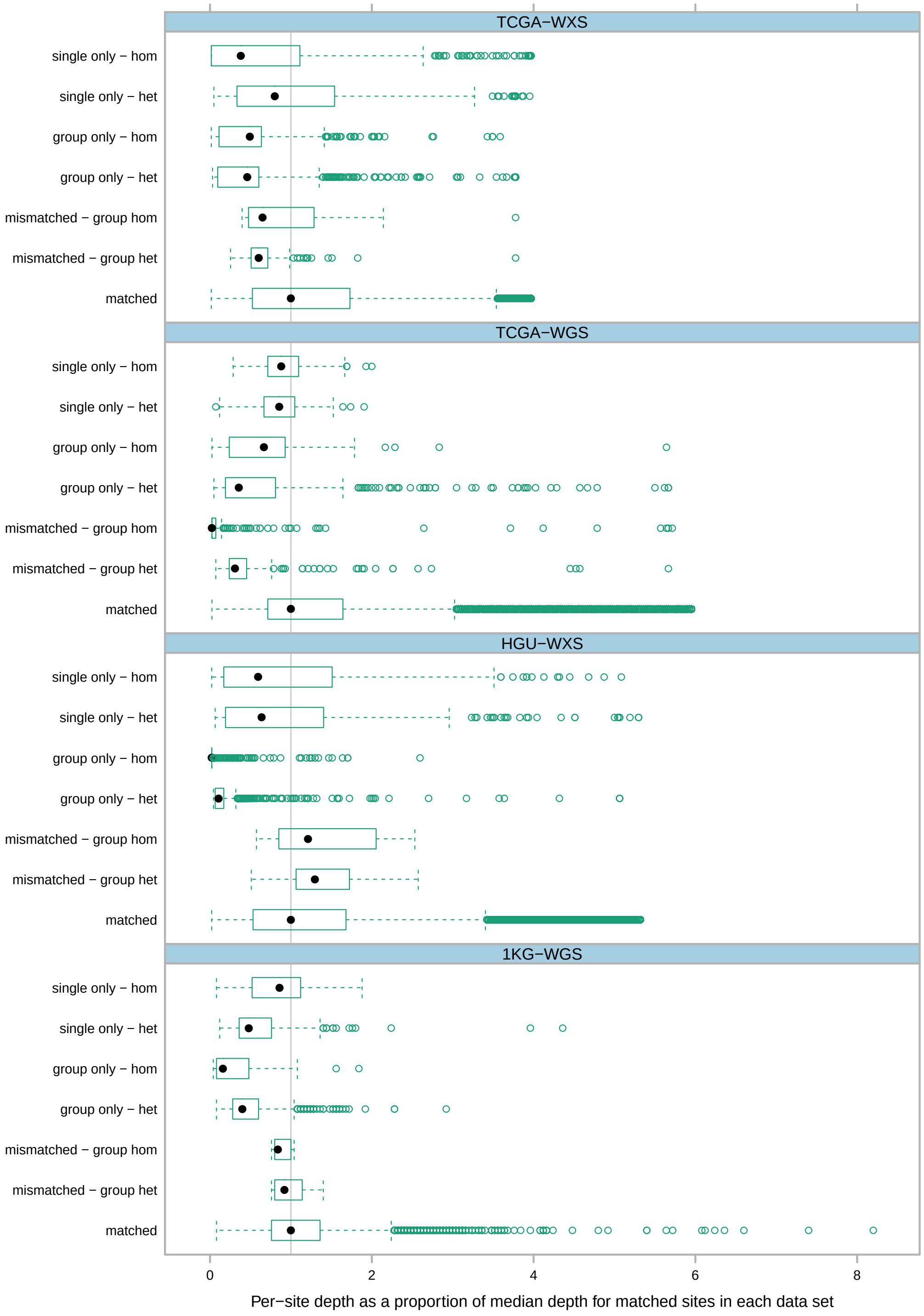

Additional File 1: Figure S10: Overall estimated sensitivity for HGMD disease-causing and disease-associated coding SNVs compared with coding regions in general.

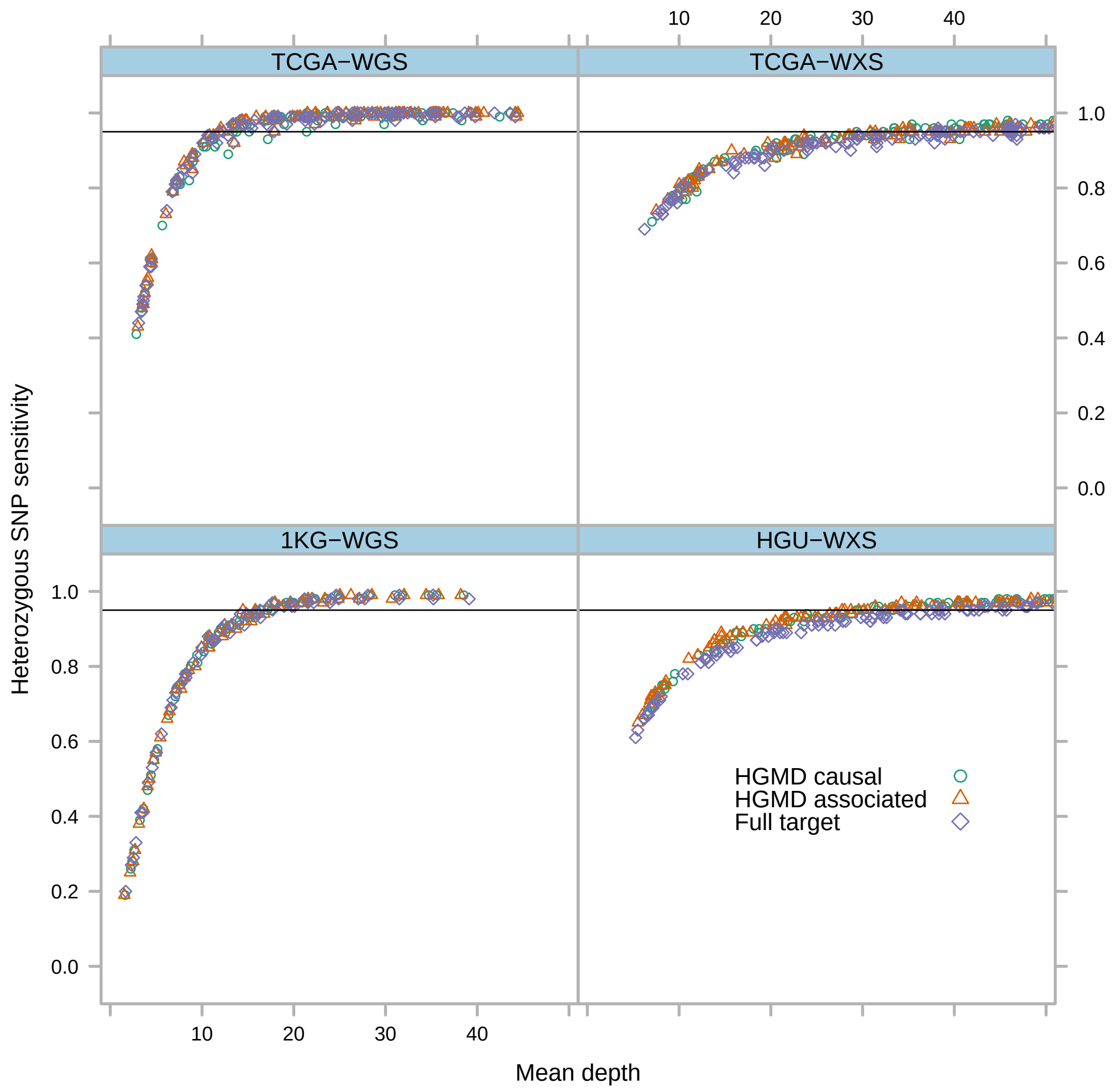

Additional File 1: Figure S11: G+C content for difficult and normal target region tiles in shared target regions.

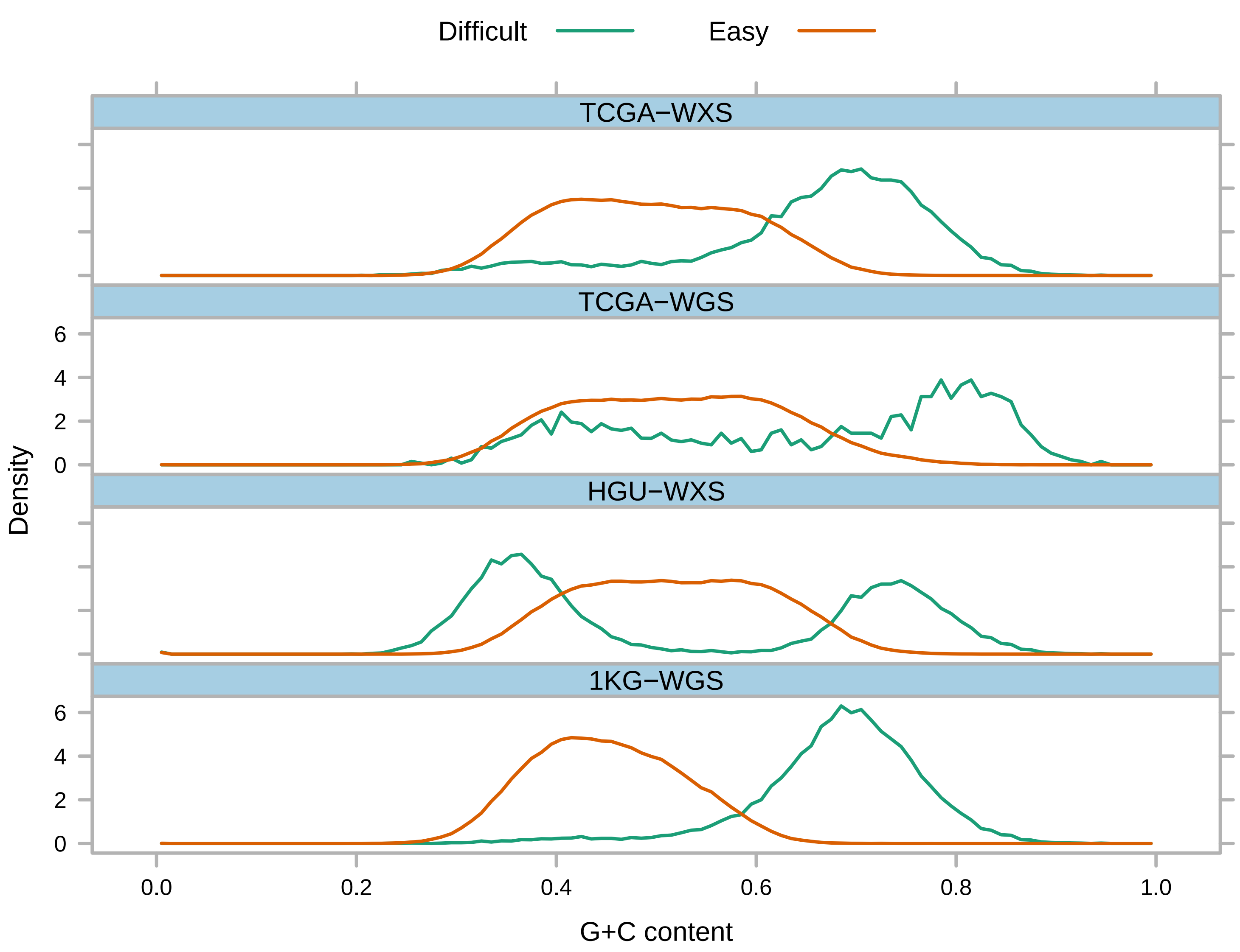

Additional File 1: Figure S12: Proportion of difficult and normal target region tiles in shared target regions overlapping repetitive elements.

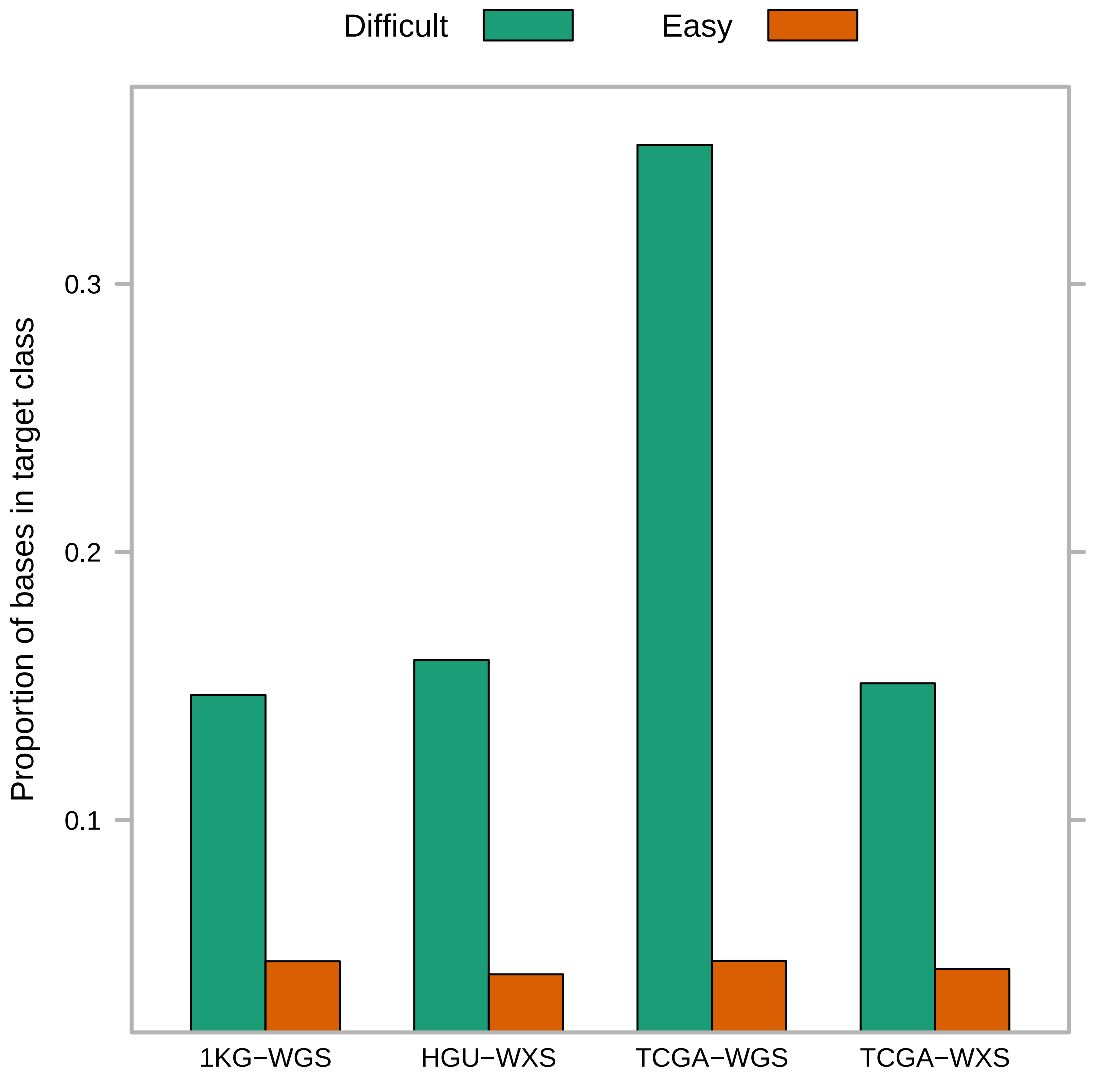

Additional File 1: Figure S13: Target region tiles classed as difficult in different data sets

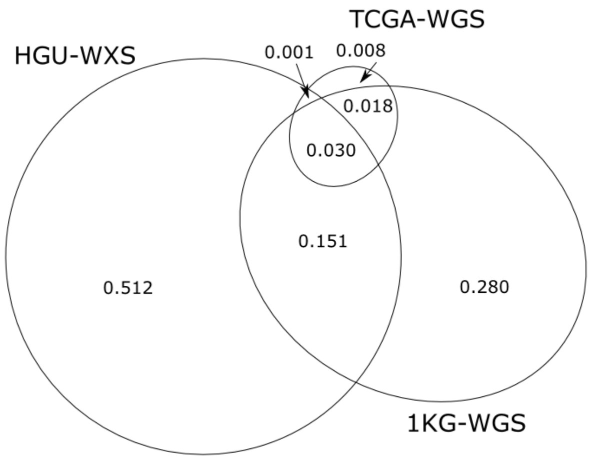

(a) Nimblegen SeqCap EZ Exome v3.

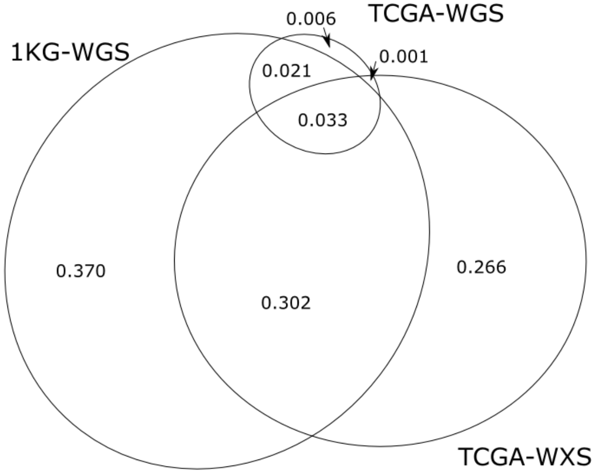

(b) Whole exome Agilent 1.1 plus boosters.

Additional File 1: Figure S14: Cost of sequencing samples to 95% overall heterozygous SNP sensitivity (1 lane of WGS, 8 samples/lane exome-seq), proportionate to per lane cost, holding the cost of exome target capture and library preparation constant. The cross-over point occurs when sequencing is 17% of the current cost.

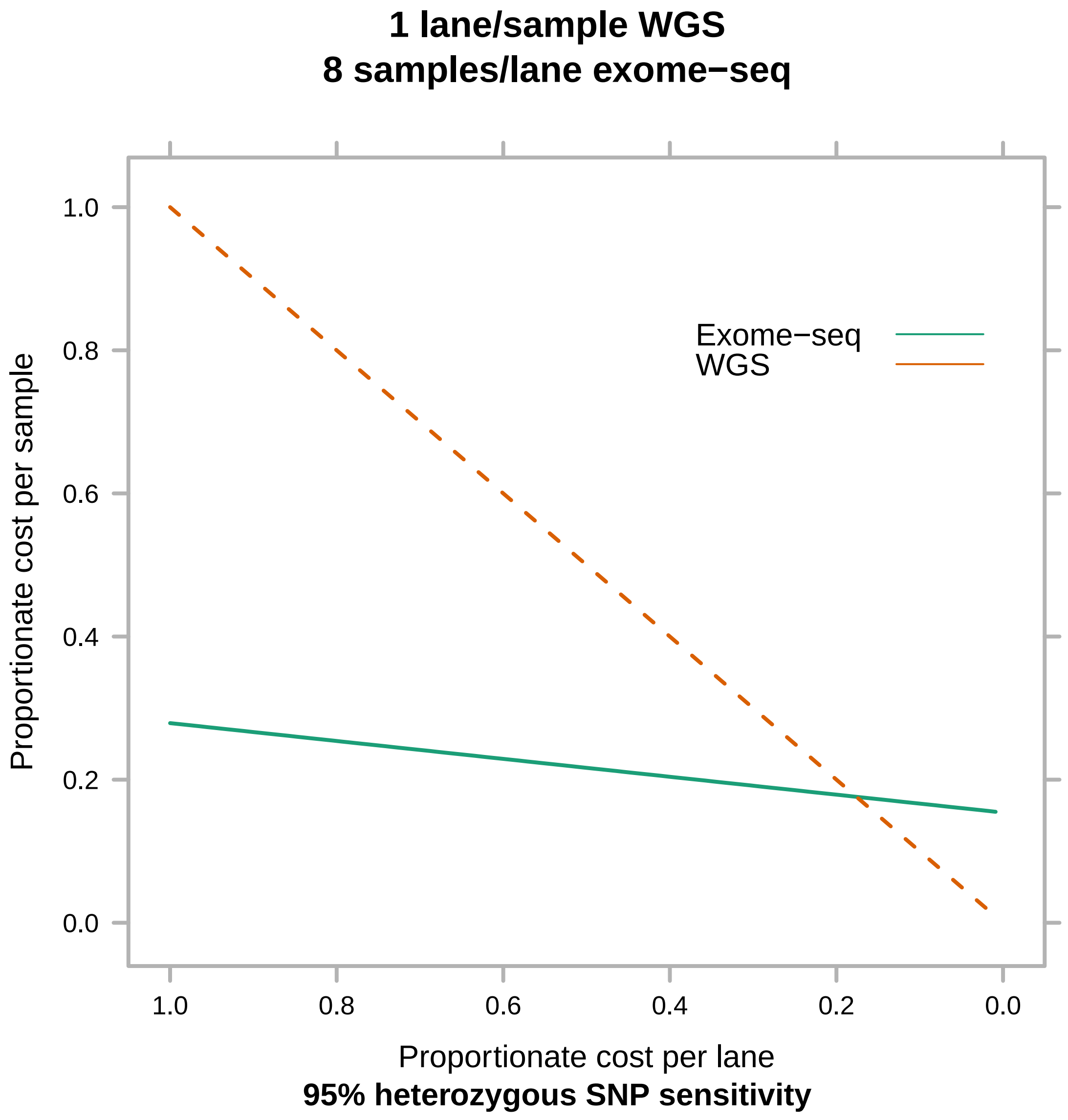

Additional File 1: Table S1: Data sources and sequencing technologies.

Data set	Samples	Sequencing technologies	Sequencer	Reads
1KG-WGS	6	Whole genome shotgun sequencing	Illumina Genome Analyzer and Genome Analyzer II	30-76bp single and paired end
HGU-WXS	13	Nimblegen SeqCap EZ Exome v3 exome capture	Illumina HiSeq 2000	98bp paired end
TCGA-WGS	10	Whole genome shotgun sequencing	Illumina Genome Analyzer II and HiSeq 2000	101bp paired end
TCGA-WXS	20	Whole Exome Agilent 1.1 plus custom boosters exome capture	Illumina Genome Analyzer II and HiSeq 2000	76bp single and paired end

Additional File 1: Table S6: Mean square difference between heterozygous SNP sensitivity curves plotted in Supplementary Figure 1.

		Target set 2	
Data set	Target set 1	SeqCap EZ Exome v3	Whole exome Agilent 1.1
1KG-WGS	Ensembl 72 coding	1.24e-04	2.27e-05
1KG-WGS	SeqCap EZ Exome v3	.	1.58e-04
HGU-WXS	Ensembl 72 coding	2.47e-05	.
TCGA-WGS	Ensembl 72 coding	1.07e-05	7.81e-06
TCGA-WGS	SeqCap EZ Exome v3	.	1.15e-05
TCGA-WXS	Ensembl 72 coding	.	1.06e-04

Additional File 1: Table S7: Genotype matching status of HapMap 3.3 sites in Ensembl 72 coding regions for full alignments of matched TCGA-WGS and TCGA-WXS samples.

Sample	Matched genotype		Mismatched genotype		WGS only		WXS only	
	Het	Hom	WGS het/WXS hom	WGS hom/WXS het	Het	Hom	Het	Hom
TCGA-05-4395	8403	6772	22	7	160	41	18	3
TCGA-05-4432	8448	6699	25	29	174	55	24	5
TCGA-05-5429	8578	6488	38	11	201	48	10	2
TCGA-06-0745	8859	6649	27	6	78	24	11	3
TCGA-64-1678	8440	6500	45	123	221	84	54	8
TCGA-CR-6470	8844	6443	25	7	155	44	5	3
TCGA-DJ-A3US	8849	6523	22	5	109	13	5	3
TCGA-DK-A3IL	8974	6398	22	11	125	40	10	4
TCGA-FK-A3SE	8827	6826	33	13	139	27	8	3
TCGA-HC-7744	8635	6566	30	28	168	35	35	3

Additional File 1: Table S8: Multiple linear regression results for poorly sequenced regions in the intersection of the two exome capture target region sets.

Data set	Alignability k-mer	Intercept	G+C content	Repeats present	Alignability	Adjusted R-squared
1KG-WGS	36	-1.15	5.91	0.92	-0.59	0.265
HGU-WXS	100	3.73	6.64	1.81	-1.18	0.086
TCGA-WGS	100	1.86	2.82	0.85	-1.59	0.150
TCGA-WXS	76	-4.74	19.40	3.00	1.47	0.171
